# Mesh-reinforced pancreaticojejunostomy versus conventional pancreaticojejunostomy after pancreaticoduodenectomy: a retrospective study of 126 patients

**DOI:** 10.1186/s12957-018-1365-y

**Published:** 2018-03-27

**Authors:** Xin Zhong, Xianfa Wang, Junhai Pan, Hepan Zhu, Lihu Gu, Zhaoqi Shi

**Affiliations:** Department of General Surgery, Sir Run Run Shaw Hospital, School of Medicine, Zhejiang University, Hangzhou, China

**Keywords:** Postoperative pancreatic fistula, Pancreaticojejunostomy, Pancreaticoduodenectomy

## Abstract

**Background:**

Pancreatic fistula is a major cause of morbidity and mortality after pancreaticoduodenectomy. The aim of this study is to compare the safety and efficacy of a newly developed technique, namely mesh-reinforced pancreaticojejunostomy, in comparison with the conventional use of pancreaticojejunostomy after undergoing a pancreaticoduodenectomy.

**Methods:**

Data was collected from regarding 126 consecutive patients, who underwent the mesh-reinforced pancreaticojejunostomy or conventional pancreaticojejunostomy, after standard pancreaticoduodenectomy by one group of surgeons, between the time period of 2005 and 2016. This data was collected retrospectively. Surgical parameters and perioperative outcomes were compared between these two groups.

**Results:**

A total of 65 patients received mesh-reinforced pancreaticojejunostomy and 61 underwent conventional pancreaticojejunostomy. There were no substantial differences in surgical parameters, mortality, biliary leakage, delayed gastric emptying, gastrojejunostomy leakage, intra-abdominal fluid collection, postpancreatectomy hemorrhage, reoperation, and the total hospital costs between the two groups. Pancreatic fistula rate (15 versus 34%; *p* = 0.013), overall surgical morbidity (25 versus 43%; *p* = 0.032), and length of hospital stay (18 ± 9 versus 23 ± 12 days; *p* = 0.016) were significantly reduced after mesh-reinforced pancreaticojejunostomy. Multivariate analysis of the postoperative pancreatic fistula revealed that the independent factors that were highly associated with pancreatic fistula were a soft pancreatic texture and the type of conventional pancreaticojejunostomy.

**Conclusions:**

This retrospective single-center study showed that mesh-reinforced pancreaticojejunostomy appears to be a safe technique for pancreaticojejunostomy. It may reduce pancreatic fistula rate and surgical complications after pancreaticoduodenectomy.

**Trial registration:**

This research is waivered from trial registration because it is a retrospective analysis of medical records.

## Background

Pancreaticoduodenectomy (PD) has, for a long time, been used as the standard surgical procedure for the treatment of patients with malignant or benign diseases of the pancreatic head or the periampullary region. Mortality in patients undergoing PD is recorded to be below 5% for general advance in surgical technique; however, postoperative morbidity remains high at 30–50% [1–3]. The main factor is postoperative pancreatic fistula (POPF), which can lead to severe secondary complications such as postoperative hemorrhages and intra-abdominal abscesses [4, 5]. Therefore, prevention and adequate treatment of POPF has always been of high priority [6]. Considerable techniques including pancreaticojejunostomy with duct to mucosa anastomosis or intussusceptions, main duct stenting and pancreaticogastrostomy have been described for safe surgical management of pancreatic remnants; however, no single method has made evident to the scientific community its superiority [7–13]. Since August 2005, our institute has attempted to reduce the frequency of pancreatic fistula (PF) by using new method termed mesh-reinforced anastomosis [14]. We have previously reported in previous studies that this technique appears to be safe, simple, and quick [14, 15]. The purpose of this retrospective study is to compare perioperative outcomes of mesh-reinforced pancreaticojejunostomy (PJ) with the conventional surgical procedure of pancreaticojejunostomy (PJ). This study was conducted by the same pancreatic team of the same institute.

## Methods

### Database

From August 2005 to November 2016, 126 patients who underwent mesh-reinforced PJ or conventional PJ after PD in our institution were included in this study. Patients’ data, including demographics, operative procedures, postoperative complications, and mortality, were retrospectively compared between 65 consecutive patients with mesh-reinforced PJ and 61 consecutive patients with conventional PJ. The perioperative management, including antibiotics, perioperative Octreotide administration, and enteral and parenteral nutrition, was the same in both groups. Definitions of pancreatic fistula (PF), postpancreatectomy hemorrhage (PPH), and delayed gastric emptying (DGE) were followed according to the International Study Group of Pancreatic Surgery (ISGPS) [16–18]. Medical morbidity was termed as conditions not related to surgical complications including cardiac, pulmonary, and renal-related complications. Follow-up results were obtained from patients’ medical records and telephone calls that were made. The diameter of the main pancreatic duct (MPD) 1 year after PD was measured on computed tomography scans and recorded. The last follow-up day was July 10, 2017. The study was approved by the Committee of Ethics of Sir Run Run Shaw Hospital of Zhejiang University. All patients signed a written informed consent acknowledging potential surgical risks.

### Operation techniques

**Fig. 1:**
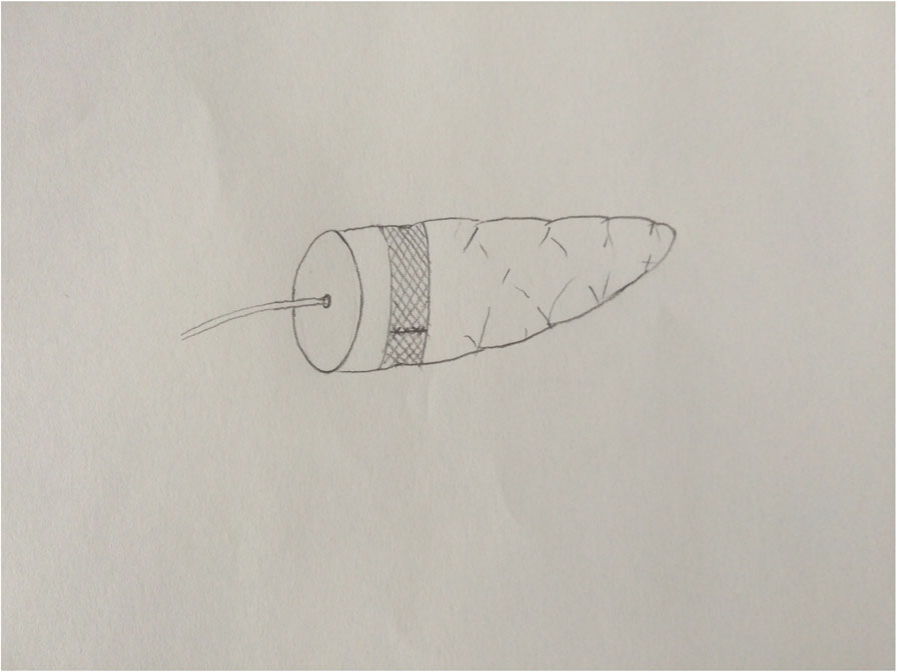
Pancreas was tightly wrapped in one circle using mesh strip. A stent tube was inserted into the pancreatic duct and fixed

**Fig. 2:**
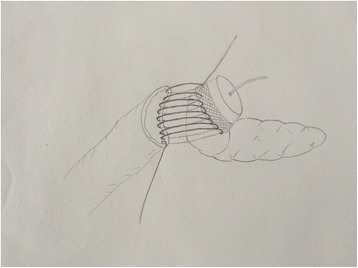
Posterior part of the jejunal stump was fixed to the inner part of the mesh in the posterior pancreatic stump using 4–0 prolene in continuous suture

**Fig. 3:**
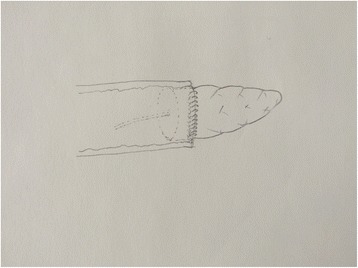
The anastomosis is complete. Mesh strip was wrapped by jejunal loop completely after prolene was fastened

In both groups, pancreatoduodenectomy (PD) was performed using the standard procedure [19]. Hemostatic management of the cut surface was done by electric coagulation or suture ligatures with 4–0 prolene stitches (Ethicon, Somerville, NJ). Our technique of mesh-reinforced PJ has been reported previously [14, 15]. In brief, pancreatic remnant was isolated 3 cm in length from the pancreatic transection. A mesh (polypropylene mesh, Ethicon, New Jersey, USA) strip of 1.5 cm in width was tightly wrapped over the pancreatic stump in one circle roughly 1.0 cm from the transection edge. The main pancreatic duct was identified, and subsequently, a stent tube was inserted (Fig. 1). The posterior part of jejunal stump and the inner part of mesh in the pancreas were anastomosed using continuous 4–0 prolene stitches (Fig. 2). The pancreatic stump invagination was performed by tightening the posterior sutures after all of the posterior stitches were completed. The continuous sutures were subsequently extended to fix the anterior part of jejunal loop and the inner part of mesh in the pancreatic stump using the same principle (Fig. 3). Conventional pancreaticojejunostomy (CPJ) with end-to-end anastomosis was performed between the pancreatic stump and the jejunum loop by two layers. The outer layer consisted of the remnant of pancreatic capsule and the seromuscular layer of the jejunum. The inner layer consisted of the pancreas and the full thickness of the jejunum. In both groups, a stent tube was placed in the main pancreatic duct.

### Statistical analyses

Statistical analysis was carried out using SPSS version 20.0 (IBM, Armonk, New York, USA). Categorical variables were presented as numbers and percentages; continuous variables were expressed as mean ± standard deviation (SD) or median (range). Categorical variables were compared using the *χ*^2^ test, or Fisher’s exact test when necessary, and continuous variables with Student’s *t* test. The risk factors of POPF were investigated by using logistic regression analysis. Parameters that were significant on univariable analysis (*p* < 0.100) and/or expected to be important clinically were included in the multivariable logistic regression model. Results were expressed as odds ratios (ORs) with 95% confidence intervals (CI). A *p* value < 0.05 was considered statistically significant.

## Results

### Patient characteristics and intraoperative data

Patient baseline parameters are shown in Table 1. There were no significant differences between mesh-reinforced PJ and conventional PJ with regard to age (*p* = 0.312), gender (*p* = 0.726), body mass index (*p* = 0.186), and the American Society of Anesthesiologists (ASA) Classification (*p* = 0.899). Intraoperative parameters are shown in Table 2. No significant differences between mesh-reinforced PJ and conventional PJ groups with regard to pathologic findings (*p* = 0.839), pancreatic texture (*p* = 0.688), and blood loss (*p* = 0.818) were observed. There was also no statistical difference between the two groups concerning operative time (359 ± 69 vs. 351 ± 63, *p* = 0.487).

**Table 1:** Patient characteristics

	mPJ group (*n* = 65)	cPJ group (*n* = 61)	*p* value
Age (years)	59.8 ± 11.2	57.8 ± 10.7	0.312
Gender (%)			0.726
Male	31 (48)	31 (51)	
Female	34 (52)	30 (49)	
Body mass index	22.6 ± 2.9	22.1 ± 1.2	0.186
Symptoms (%)			
Jaundice	31 (48)	28 (46)	0.840
Pain	15 (23)	13 (21)	0.812
Medical risk			
ASA score	2 (1–3)	2 (1–3)	0.899
Cardiopulmonary disease (%)	14 (22)	13 (21)	0.975
Diabetes (%)	12 (18)	8 (13)	0.412

*mPJ* mesh-reinforced pancreaticojejunostomy, *cPJ* conventional pancreaticojejunostomy

**Table 2:** Diagnosis and operative data

	mPJ group (*n* = 65)	cPJ group (*n* = 61)	*p* value
Pathology (%)			0.839
Pancreatic cancer	18 (28)	13 (21)	
Bile duct cancer	22 (34)	24 (39)	
Ampullary cancer	8 (12)	6 (10)	
Duodenum cancer	12 (18)	11 (18)	
Other	5 (8)	7 (11)	
Pancreatic texture (%)			0.765
Hard	39 (60)	35 (57)	
Soft	26 (40)	26 (43)	
Main pancreatic duct (%)			0.975
≤ 3 mm	51 (78)	48 (79)	
> 3 mm	14 (22)	13 (21)	
Operative time (min)	359 ± 69	351 ± 63	0.487
Blood loss (mL)	606 ± 218	626 ± 224	0.818

*mPJ* mesh-reinforced pancreaticojejunostomy, *cPJ* conventional pancreaticojejunostomy

### Major outcomes and complications

Table 3 describes the postoperative complications and economic outcomes. The overall in-hospital mortality was recorded as 4.0% (5 of 126). One fatality was recorded in the mesh-reinforced PJ group, and four fatalities occurred in the conventional PJ group. The cause of death was intraperitoneal hemorrhage in mesh-reinforced PJ group (*n* = 1). And in conventional PJ group, the causes of death were multiorgan failure (*n* = 2), intraperitoneal abscess (*n* = 1), and intraperitoneal hemorrhage (*n* = 1). There were no substantial differences in mortality between the two groups (*p* = 0.197). For patients who survived the operation, seven patients required reoperation due to pancreatic anastomosis leakage (*n* = 3), gastrojejunostomy leakage (*n* = 2), or intraperitoneal hemorrhage (*n* = 2). The reoperation rate was 3% (2 of 65) in the mesh-reinforced PJ group and 8% (5 of 61) in the conventional PJ group, with no significant differences (*p* = 0.262). Postpancreatectomy hemorrhage (PPH) was observed in nine patients. All mentioned cases occurred more than 24 h after PD and originated from the peripancreatic region. The treatment for PPH included blood transfusion (9/9), arterial embolization (4/9), and re-laparotomy for severe PPH (2/9). Postpancreatectomy hemorrhage revealed no substantial difference between the two groups (*p* = 0.454). Delayed gastric emptying (DGE), biliary leakage, and gastrojejunostomy leakage, as well as intra-abdominal fluid collection, also showed no significant differences between the two groups (Table 3). The incidence of POPF was significantly different between mesh-reinforced PJ and the conventional PJ group (15 vs. 34%, respectively; *p* = 0.013) (Table 3). Eleven cases of pancreatic fistula of grade B were managed by antibiotics and total parenteral nutrition, and all recovered well after conservative treatment. Treatment for grade C pancreatic fistula included percutaneous drainage (4/4) and re-laparotomy (3/4). One case of grade C in the mesh-reinforced PJ group was treated by re-laparotomy and ultimately recovered well. Two cases of grade C in the conventional PJ group received re-laparotomy and died from POPF-induced sepsis and bleeding. Total surgical morbidity (25 versus 43%; *p* = 0.032) and postoperative length of hospital stay (18 ± 9 days vs. 23 ± 12 days; *p* = 0.016) were significantly different between mesh-reinforced PJ and conventional PJ groups (Table 3). There was no substantial difference in the total hospital costs (106,265 ± 1231 RMB vs. 114,265 ± 1349 RMB; *p* = 0.142) between mesh-reinforced PJ and conventional PJ groups (Table 3). Forty-one cases in the mesh-reinforced PJ and 45 cases in the conventional PJ group received computed tomography scans in a yearly follow-up (Table 4). No statistical differences between the two groups concerning dilated pancreatic duct were observed (*p* = 0.578).

**Table 3:** Postoperative complications

	mPJ group (*n* = 65)	cPJ group (*n* = 61)	*p* value
Mortality (%)	1 (2)	4 (7)	0.197*
Pancreatic fistula grade (%)			0.014
Overall	10 (15)	21 (34)	
A	8 (12)	8 (13)	
B	1 (2)	10 (16)	
C	1 (2)	3 (5)	
Delayed gastric emptying (%)			0.653*
A	2 (3)	2 (3)	
B	2 (3)	4 (7)	
Intra-abdominal fluid collection or abscess (%)	5 (8)	10 (16)	0.132
Postpancreatectomy hemorrhage (%)			0.454
B	2 (3)	5 (8)	
C	1 (2)	1 (2)	
Biliary leakage (%)	3 (5)	4 (7)	0.711*
Gastrojejunostomy leakage (%)	2 (3)	3 (5)	0.673*
Reoperation (%)	2 (3)	5 (8)	0.262*
Overall surgical morbidity (%)	16 (25)	26 (43)	0.032
Medical morbidity (%)	8 (12)	7 (11)	0.910
Postoperative hospital stay (d)	18 ± 9	23 ± 12	0.016
Total hospital costs (RMB ¥)	106,265 ± 1231	114,265 ± 1349	0.142

*mPJ* mesh-reinforced pancreaticojejunostomy, *cPJ* conventional pancreaticojejunostomy

*Fisher’s exact test

**Table 4:** Main pancreatic duct changes 1 year after PD

	mPJ group (*n* = 41)	cPJ group (*n* = 45)	*p* value
Pancreatic duct before operation (mm)	2.2 ± 2.8	2.7 ± 3.2	0.475
Pancreatic duct after operation (mm)	3.6 ± 4.1	3.4 ± 3.9	0.578

*mPJ* mesh-reinforced pancreaticojejunostomy, *cPJ* conventional pancreaticojejunostomy

### Risk factors for development of pancreatic fistula

Predictors of PF for all patients are shown in Table 5. Factors significantly increasing the risk of PF by univariate logistic regression analysis included soft pancreatic texture, ampullary or duodenal disease, and type of conventional pancreaticojejunostomy (*p* < 0.05). A multivariate logistic regression analysis revealed that the independent factors that were highly associated with PF were soft pancreatic texture and type of conventional pancreaticojejunostomy (*p <* 0.05).

**Table 5:** Univariate and multivariate logistic regression analysis of risk factors for pancreatic fistula

	Univariable *p*	Multivariable *p*	Odds ratio	95% CI
Age	0.814			
Gender	0.150			
Jaundice	0.424			
Abdominal pain	0.651			
Type of anastomosis				
mPJ	0.013	0.007	0.223	0.076–0.658
cPJ			1	
Pancreatic texture				
Hard	< 0.001	< 0.001	0.029	0.007–0.111
Soft			1	
Main pancreatic duct				
≤ 3 mm	0.183			
> 3 mm				
Operative time	0.693			
Blood loss	0.457			
Pathology	< 0.001	0.548		
Pancreas	0.082	0.105		
Bile duct	< 0.001	0.502		
Ampulla	0.093	0.507		
Duodenum	0.001	0.829		
Other	/	/		

*mPJ* mesh-reinforced pancreaticojejunostomy, *cPJ* conventional pancreaticojejunostomy

## Discussion

Despite the remarkable progress in surgical technique and perioperative care during the last decades, the pancreatic-enteric anastomosis remains the “Achilles heel” of PD. Although more than 80 different methods have been described for safe surgical management of the pancreatic-enteric anastomosis, none have been proven to be superior techniques over others, and consequently became widely accepted [20]. Because most of these surgical methods include stitches that penetrate through the pancreatic parenchyma and the soft pancreatic tissue, it makes the pancreas vulnerable to the formation of PF [21, 22]. Peng and coworkers [23] performed a comprehensive three-layer invagination anastomosis, called binding anastomosis, to protect the pancreatic anastomosis from PF. Remarkably, a 0% rate of PF has been reported using this technique. However, this procedure includes complex and troublesome maneuvers. To effectively prevent the PF, we have designed a new technique, namely “mesh-reinforced pancreaticojejunostomy.” This technique using single-layer continuous suturing is far less complex when compared with the binding pancreaticojejunostomy. In the present study, there is no significant difference between mesh-reinforced PJ and conventional PJ in regard to operative time (*p* > 0.05). This new technique of mesh-reinforced pancreaticojejunostomy is favored for patients with a soft pancreatic remnant. Mesh around the pancreatic remnant provides a safe anchor site for the suture, which is particularly suitable for soft pancreatic parenchyma to avoid anastomotic dehiscence. In the present study, the rate of overall PF was recorded to be 15% in the mesh-reinforced PJ group and 34% in the conventional PJ group with a significant difference (Table 3).

Several previous studies have evaluated risk factors of pancreatic fistula after pancreatic-enteric anastomosis. These risk factors include age, prolonged jaundice, and intraoperative blood loss, all of which have been associated with an increased risk of PF [24, 25]. In the current study (Table 5), demographic factors, such as age, gender, and prolonged jaundice were not statistically associated with pancreatic fistula. Intraoperative parameters, such as soft pancreatic texture and pathology diagnoses, were found to positively correlate with the risk of PF on univariate analysis (*p* < 0.05). However, pathological diagnosis failed to maintain its statistical significance in the multivariate model. In the stepwise multivariate logistic regression analysis (Table 5), independent factors influencing PF rates were the texture of the organ and type of conventional pancreaticojejunostomy (*p <* 0.05). Thus, these results were similar with a number of studies [26–28], which reported that a soft pancreatic remnant is more likely to develop PF.

To identify new surgical techniques that can substantially lead to decrease mortality rates is a challenging task due to the fact that operative mortality in patients undergoing PD is already low. Therefore, the length of postoperative hospital stay comes to be an important representation of patients’ condition and surgical outcome. A shorter length of postoperative hospital stay is considered a predictor of less-invasive surgical procedures and less cost of medical expense. Our data demonstrates that length of hospital stay was significantly reduced in mesh-reinforced PJ group (18 ± 9 vs 23 ± 12 days; *p* = 0.016). This shows mesh-reinforced pancreaticojejunostomy developed a fast-track postoperative course. The possible explanation is that surgical complications and POPF rate were significantly less common in the mPJ group (*p* < 0.05 Table 3). The occurrences of POPF and surgical complications contribute to an increased length of postoperative hospital stay. The cost of mesh increased hospital cost, but there was no significant difference in total hospital cost between the two groups (*p* > 0.05 Table 3). We found that the length of postoperative hospital stay, surgical complications, and POPF rates were significantly less common in the mPJ group (*p <* 0.05 Table 3) which reduced the total hospital cost.

The advantages of mesh-reinforced PJ [14, 15] are as follows: Firstly, mesh provided a safe anchor site for the suture to avoid laceration of anastomotic and postoperative bleeding; secondly, mesh compression to pancreatic tissue minimized the chance of pancreatic leakage and bleeding; thirdly, mesh was thought to stimulate growth of fibroblast and enhance the anastomotic healing process. We considered that these advantages of mesh decreased pancreatic fistula rate, hospital stay, and the complication rate.

Theoretically, the use of mesh has potential disadvantages. As an implanted foreign body, mesh may increase the risk of intra-abdominal infection. However, our data showed there was no statistical difference between the two groups concerning occurrence of abdominal infections (*p* > 0.05). We think it may be because of the fact that the mesh was completely wrapped by the jejunal loop during the procedure. On the other hand, the mesh had contractility, which may result in pancreatic atrophy or pancreatic duct dilation. Prolene-Mesh had a contractility of around 20% [29]. We used polypropylene-mesh reinforcement in 10 pigs in an animal experiment, which showed that the pancreatic duct was dilated, and the mesh was rejected 3 months after mesh-reinforced pancreatojejunostomy in all experimented pig subjects. However, the pancreatic duct of the pig was too fine to have a stent tube placed inside. Our data showed there was no statistical difference between the two groups concerning dilated pancreatic duct at the 1-year follow-up, demonstrating that the stent tube withstood compression by the mesh strip. Unfortunately, the anastomotic patency and postoperative pancreatic function was not examined in our study.

## Conclusions

In conclusion, this retrospective single-center study showed that mesh-reinforced pancreaticojejunostomy appears to be a simple and safe technique for pancreaticojejunostomy. It provided a safe anchor site for the suture, which was especially suitable for the soft and fragile pancreatic texture to avoid laceration of anastomotic sutures and prevent pancreatic leakage. It should be applicable to all types of pancreatic remnant. Our study was a retrospective analysis of medical records. It should be mentioned that it has all the disadvantages that are associated with any retrospective series. A prospective randomized control study is necessary to confirm the efficacy of this procedure in future.

## Abbreviations

ASA: American Society of Anesthesiologists
CI: Confidence intervals
DGE: Delayed gastric emptying
ISGPS: International Study Group of Pancreatic Surgery
MPD: Main pancreatic duct
PD: Pancreaticoduodenectomy
PF: Pancreatic fistula
PJ: Pancreaticojejunostomy
POPF: Postoperative pancreatic fistula
PPH: Postpancreatectomy hemorrhage
SD: Standard deviation
